# A review of key terminology and definitions used for birth defects globally

**DOI:** 10.1007/s12687-023-00642-2

**Published:** 2023-04-24

**Authors:** HL Malherbe, B Modell, H Blencowe, KL Strong, C Aldous

**Affiliations:** 1 Centre for Human Metabolomics, North-West University, North-West Province, Potchefstroom, South Africa; 2 Rare Diseases South Africa NPC, The Station, 63 Peter Place, Bryanston, Johannesburg, 2021 Gauteng South Africa; 3 University College London, London, UK; 4 Centre for Maternal, Adolescent, Reproductive and Child Health, London School of Hygiene and Tropical Medicine, London, UK; 5 Department of Maternal, Newborn, Child and Adolescent Health and Aging, Health, World Health Organization, Geneva, Switzerland; 6 School of Clinical Medicine, University of Kwa-Zulu Natal, Durban, South Africa

**Keywords:** Congenital disorders, Congenital anomalies, Birth defects, Terminology, Definitions, Burden of disease

## Abstract

Birth defects, also known as congenital disorders, are a significant health issue impacting at least five million births annually worldwide. For policymakers to mount a relevant healthcare response to care for those affected, the burden of disease of these conditions must be quantified. Estimates of the contribution of birth defects to under-5 child mortality and morbidity are generated by several groups globally. These estimates often differ, causing confusion for policymakers. While some differences may be attributed to the data sources and methods used, much is due to a lack of clarity in the terminology used for the group of disorders classed as “congenital”. This study aimed to gain insight into the diversity of terms and definitions for birth defects, including those used routinely by relevant international/national organisations and in the peer-reviewed literature. This two-part study included (1) scoping review of peer-reviewed literature to identify terms and definitions in use for birth defects and (2) review of key websites and grey literature to identify terms and definitions used. The results of this study indicate a wide variety of terms being used, often interchangeably and undefined, in peer-reviewed publications, on institutional websites and related literature. This suggests a lack of clarity related to terminology and sets the scene for further discussion, recommending that the community of practice working on birth defects comes to a consensus on standard terminology and definitions for global uptake and implementation. Such standardisation will facilitate a common understanding of the burden of these disorders globally, regionally and within countries so that action can be taken to support affected children and their families.

**Supplementary Information:**

The online version contains supplementary material available at 10.1007/s12687-023-00642-2.

## Introduction

The heterogenous collective of congenital disorders (also known as birth defects) includes a diverse range of conditions present from birth as well as minor conditions (e.g. polydactyly) that do not pose a significant health issue. Some disorders, such as cleft lip and spina bifida, are obvious at birth while other serious conditions, such as congenital heart defects and inborn errors of metabolism, are less easily detected. Other conditions may only manifest later in life, for example, Huntington’s disease. The aetiology of these conditions varies, ranging from genetic or partially genetic, sporadic, or environmental causes. Serious congenital disorders, defined as those resulting in death or disability in the absence of intervention, are an important cause of early death and life-long disability (Christianson et al. 2006; Modell et al. 2016). The collective of congenital disorders represents a significant health issue impacting at least five million births annually across the globe (World Health Organization 2006, Modell et al. 2016).

While congenital disorders impact all populations, their occurrence varies across and between populations, with the majority of disorder-related deaths occurring in low- and middle-income countries (LMIC) (Christianson et al. 2006; Perin et al. 2023). While some conditions vary in their occurrence geographically, the prevalence of others is predictable between populations (Moorthie et al. 2018). Their birth prevalence is affected by various factors, including the degree of development of healthcare infrastructure and capacity (e.g. antenatal and perinatal care, vaccination programmes, diagnostic capacity, availability of primary and secondary preventative measures), prevalence of risk factors (poverty, maternal age at conception, frequency of consanguineous unions) and environmental exposures. Factors such as protection of carriers of haemoglobin disorders and glucose-6-phosphate dehydrogenase (G6PD) deficiency against death from malaria^1^ must also be considered (Christianson et al. 2006).

While under-5 survival has improved over the past two decades globally, the proportion of child deaths due to congenital disorders has increased, from 4.6% of under-5 deaths in 2000 to 7.6% in 2019 (Perin et al. 2022, 2023). This is most prominent in countries with the lowest under 5 mortality rates, where congenital disorders account for 20–30% of under-5 deaths (Draper et al. 2021; Ely and Driscoll 2021; Perin et al. 2023). This includes many high-income countries (HIC) that have completed epidemiological transition (Omran 1971). The proportion of child deaths due to congenital disorders is also set to increase in low-income countries, as parallel and persisting infectious diseases (e.g. acute respiratory infections, diarrhoea and malaria) are better managed and eradicated (Christianson and Modell 2004; Kahn et al. 2007; Malherbe et al. 2015; Kabudula et al. 2017).

Estimates of the contribution of congenital disorders to these adverse outcomes (mortality and morbidity) generated by several groups globally differ, causing confusion for policymakers. While some differences are attributed to the use of different data sources and methods, much of the difference is due to the use of different terminology for the conditions classed as “congenital”. This article addresses the need for an agreed terminology related to congenital disorders, to enable professional and public perception of the true scale of the problem.

### Why is consensus on terminology important?

Early, optimal services and interventions can prevent death and mitigate disability for 70% of those affected by congenital disorders (Czeizel et al. 1993; Christianson and Modell 2004, World Health Organization 2006). However, the planning and provision of appropriate health services for those affected requires detailed, accurate and reliable epidemiologic data to quantify this burden of disease in different settings (World Health Organization 1996, 2006). A first step to obtaining such agreement is to clearly define and classify the collective and conditions for inclusion and review in the burden of disease (World Health Organization 1996, 2006). Consensus on global, uniform definitions and terminology related to congenital disorders and consistent implementation of these agreed terms is currently lacking. When the concept of community genetics^2^ was introduced in 1992, it was recognised that “to create the scientific basis that we need,—principles and methods must be clearly enunciated, gain wide acceptance and be applied in practice” (Modell 1992). This applies today for the terms used to describe the collective, and sub-sets, of congenital disorders.

Several key challenges arise from the continued use of diverse terminology and differences in the range of conditions included in the collective by stakeholders. Perhaps of greatest concern is the impact of this ambiguity on the perception of the burden of disease by policymakers. For example, congenital disorders are defined by the World Health Organization (WHO) as “any potential pathological conditions arising before birth, whether they are evident at birth or become manifest later in life” (World Health Organization 1985, 2000, 2006, World Health Assembly 2010)—but undefined sub-sets of disorders representing only a portion of this disease burden are regularly presented by stakeholders and may be misinterpreted as the totality of the burden due to the use of non-synonymous terms (Malherbe et al. 2016; Modell et al. 2016, 2018). The estimated global live birth prevalence of congenital *anomalies* (structural anomalies only) is 22 per 1 000 live births, but this is often taken to represent total congenital *disorders* (structural *and* functional anomalies), which affect at least 36 per 1 000 births (Czeizel and Sankaranarayanan 1984; Christianson et al. 2006; Modell et al. 2016). In a population experiencing a million births per year, this equates to 20 000 births affected by congenital anomalies versus 39 000 affected by the larger collective of congenital disorders (Statistics South Africa 2021). If policymakers perceive structural anomalies to represent the total burden of congenital disorders, 19 000 affected births remain unaccounted for and underserved by appropriate health care in the country. This issue is relevant to health care in all populations but is most marked in extremely resource-constrained populations in LMIC (Christianson et al. 2006). It is vital that the congenital disorder-related burden of disease is accurately and comprehensively reported in these countries (Malherbe et al. 2015, 2016).

### Current terminology

**Fig. 1:**
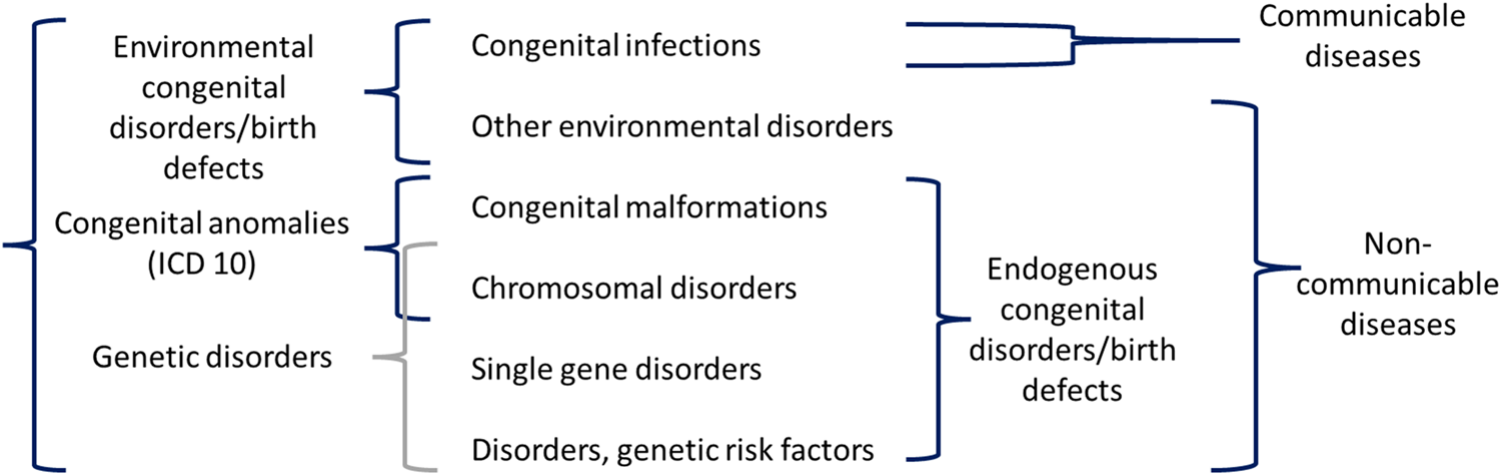
A breakdown of the WHO definition of congenital disorders showing how the main disorder groupings are bundled for different professional purposes and possibilities for confusion in the absence of clear definitions. Reproduced with author permission from Modell et al. 2016. Epidemiological methods in community genetics and the Modell Global Database of Congenital Disorders (MGDb). https://discovery.ucl.ac.uk/id/eprint/1532179/

Comprehensive evaluation and consensus of terms and definitions related to congenital disorders has not been undertaken in the scientific literature and has received limited attention at international fora (Brisson 2000, Malherbe et al. 2016, Malherbe et al. 2018, Darlison and Malherbe 2020). The 2006 March of Dimes (MOD) Global Report on Birth Defects (Christianson et al. 2006) made use of the Modell Global Database (MGDb), to produce country-level estimates for early-onset, congenital disorders. Later, the MGDb was further developed at the request of WHO/MOD to expand these estimates (World Health Organization 2006, Modell et al. 2016). The MGDb developed a glossary of definitions to explain the ill-defined and often overlapping terms (Fig. 1).

Single gene disorders are excluded from the congenital anomaly grouping in Fig. 1. Most single gene disorders are also considered rare diseases. While individually rare, collectively, rare diseases are common, with over 7 000 characterised to date, accounting for a third of congenital disorders (Haendel et al. 2020). This descriptive, desktop study was designed to gain insight into the diversity of terms and definitions currently in use for congenital disorders, including those used routinely by relevant international and national organisations and in the peer reviewed literature, and set the scene for further discussion aiming to achieve consensus on standardised terminology and definitions for global uptake and implementation.

## Method

This study was undertaken in two parts: (1) review of peer-reviewed medical literature using components of scoping review methodology to identify terms and definitions in use for congenital disorders and (2) review of key websites and grey literature to identify terms and definitions used.

### Peer-reviewed literature

The preliminary steps of a scoping review as described by Arksey and O’Malley (2005) were followed. This included the following:

- *Term identification:* development of list of key terms and relevant synonyms, including MeSH terms (Table 1).
- *Eligibility criteria:* Peer-reviewed articles of all types (i.e. conference-proceedings, systematic and other reviews, clinical trials, case studies) excluding books and book sections. Published between 2002 and 2022 to differentiate terms used prior- and post-2006 consensus meeting (World Health Organization 2006). Articles published in English only (due to limited resources) were searched on title and abstract (tiab).
- *Information sources:* a single electronic database, PubMed, was used due to its broad clinical focus. PubMed searches were undertaken on 17 and 23 August 2022.
- *Search terms:* terms, including MeSH terms, were compiled by two authors (HM and CA) following preliminary searches. A total of 251 terms and phrases were identified. An individual search (on title and abstract—tiab) was undertaken for each of the 251 terms using quotation marks for terms of more than one word, and wild card (*) was used with root words to retrieve all term suffixes and variations, e.g. “Birth defect*” [tiab] and “Congenital disorder*” [tiab]. For examples of the terms searched, see Table 1, and for a complete list of the Boolean strings in all PubMed searches and results, see Supplementary File 1.
- *Screening and study selection:* individual search results were imported into Endnote reference manager software (version 20, Clarivate). Duplicates were removed from results of each individual term search.
- *Data abstraction and data synthesis*: due to the high volume of searches (251) and results, a sample of 20 articles from each search term that returned > 100 results were identified using an online, random number generator (www.random.org) after sorting records by year and alphabetically by surname. Results of three specific search terms (congenital anomal/anomalies, birth defects and congenital disorders) were further evaluated following earlier WHO recommendations (World Health Organization 2006). The full text of sample articles was coded and evaluated using NVivo software (QSR International Pty Ltd, March 2022 release 1.6.2) to identify the definition or context of the terms use. For each randomly selected sub-set of 20, the 10 articles with the highest number of different terms coded during analysis were described in further detail.

**Table 1:** Examples of individual search terms searched in PubMed on title and abstract. See Supplementary File 1 for list of all 251 search strings

Root word	Additional term(s)
Birth	anomal*, defect*, risk*
Chromosom*	abnormal*, anomal*, defect*, disorder*, mutation*, syndrome*
Congenital	abnormal*, anomal*, defect*, deform*, disease*, disorder*, malform*, syndrom*
Complex	abnormal*, anomal*, defect*, deform*, disease*, disorder*, malform*, mutation*, risk*, syndrom*
Endogenous	risk*
Environment*	risk*
Fetal/foetal	anomal*, abnormal*, defect*, disease*, disorder*, malform*, risk*, syndrome*,
Genetic	abnormal*, anomal*, defect*, disease*, disorder*, malform*, mutation*, predispos*, risk*, syndrom*
Hereditary	disease*, disorder*, mutation*, predispos*, risk*, syndrome*
Inherited	abnormal*, defect*, disease*, disorder*, mutation*, predispose*, risk*, syndrome*, genetic disease*, genetic disorder*, genetic dysmorph*, genetic mutation*, genetic predispos*
Monogenic	Defect*, disease*, disorder*, mutation*, syndrome*
Multifactorial	disease*, disorder*, risk*, syndrome*
Polygenic	disease*, disorder*, risk*
Single gene/single-gene	defects*, disorders*
MeSH terms	“Congenital Abnormalities”[Mesh] OR “Genetic Diseases, Inborn”[Mesh]

### Website review

A list of key international organisations primarily involved in the field of congenital disorders were identified through the application of the combined authors knowledge and a Google search (https://www.google.com/) undertaken in Johannesburg, South Africa, in August 2022 and January 2023 using three specific terms: birth defects, congenital disorders and congenital anomalies. Key international organisations identified by the authors found in the first 20 results returned for each search term were included. To better understand national-level use of terminology, searches were undertaken for a limited number of English-speaking high-income countries (Australia, Canada, New Zealand, the UK and the USA). Examples from different stakeholder groups were sought (i.e. government, registries and patient support organisations) in these countries, where available. Terms relating to congenital disorders on the websites included from the Google searches were recorded together with their definition (when provided) and evaluated.

## Results

### Peer-reviewed literature

**Fig. 2:**
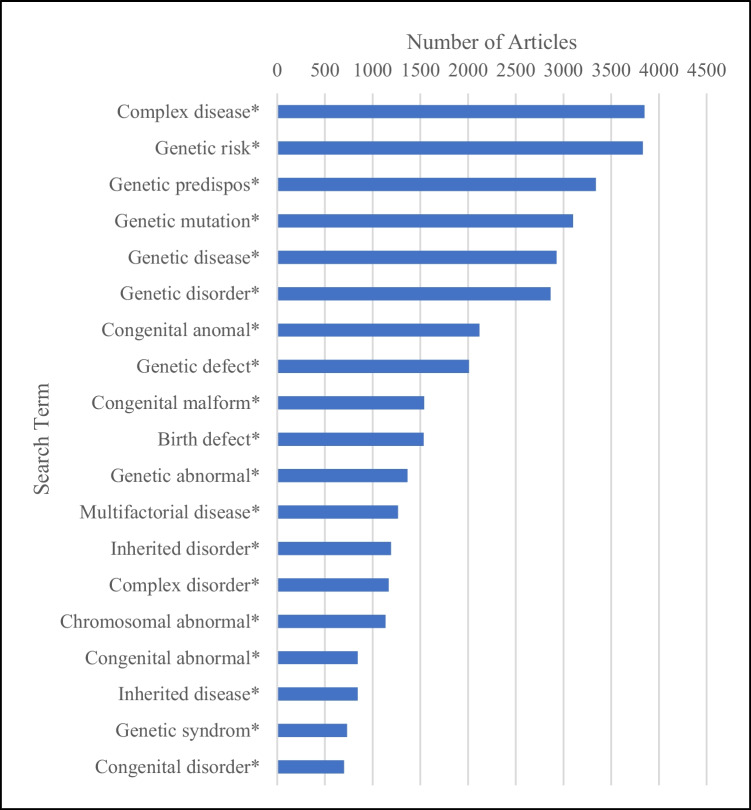
Top 20 terms resulting from individual PubMed searches from 2002 to 2022

Of the 251 PubMed searches undertaken, over a third (*n* = 88, 35%) returned zero articles meeting the inclusion criteria. A fifth of searches (*n* = 52, 21%) returned results for over 100 articles, and of these, the top 20 search terms (i.e. returning the most articles) are presented in Fig. 2. Duplicates (*n* = 705) were removed for synonymous terms, including single gene/single-gene defect* (*n* = 141), single gene/single-gene disorder (*n* = 354) and single gene/single-gene mutation (*n* = 207). Duplicate articles recorded between searches were not deleted since this indicated the use of multiple terms in one article.

The top five terms identified were “complex disease*” (*n* = 3848 articles), “genetic risk*” (*n* = 3830), “genetic predispose*” (*n* = 3338), “genetic mutation*” (*n* = 3103) and “genetic disease*” (*n* = 2928).^3^ The first four of these terms were excluded from further analysis as they are broadly used across different fields of medicine and not solely applicable to congenital disorders.

Of the three specific terms—congenital anomaly/anomalies, birth defects and congenital disorders—further evaluated, “congenital anomaly/anomalies” returned 2118 articles, ranking 3^rd^ overall (following abovementioned exclusions). Both “birth defects” and congenital disorders” were included in the top 20 searches, returning 1533 and 699 articles and ranking 6^th^ and 15^th^ respectively.

**Fig. 3:**
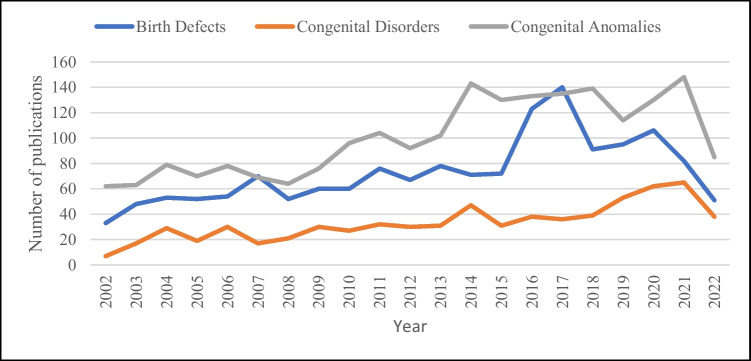
Number of publications in PubMed searches for three key search terms: “Birth defect*”, “congenital disorder*” and “congenital anom*” searching on title and abstract [tiab] from 2002 to 2022

Figure 3 shows the number of peer-reviewed articles found in the 20-year study period in searches using the terms birth defects, congenital disorders and congenital anomalies. While there is an overall increase in the number of articles found for all three terms over the period, particularly from 2013, congenital anomalies resulted in the most articles, both prior to and after 2006 (Fig. 3). Further analysis of the sub-set of articles found for these three search terms indicate that clear definitions of these terms are rarely provided, and in many cases, these three terms are used inconsistently and often synonymously with other terms (Table 2). Of the 20 articles detailed in Table 2, 17 (70%) did not define any terms used and six (30%) defined some terms used but not all.

**Table 2:** Range of terms and definitions identified in articles resulting from the three key search terms (birth defect*, congenital disorder* and congenital anomal*)^

Search term	Authors	Terms used in articles	Definitions
Birth defect(s)	Bendixen et al. (2021)	Birth defect, congenital anomaly, monogenic/monogenic syndromes, defect, malformation, structural anomaly	Undefined
	Uguz (2020)	Congenital anomalies, birth defect(s), congenital malformation, minor/major congenital anomalies, congenital heart defects (CHD), congenital abnormalities	Undefined
	Reefhuis et al. (2015)	Birth defect, chromosomal disorders and monogenic disorders	Undefined Undefined
	Donner et al. (2010)	Birth defect, congenital abnormalities	Undefined
	Ladd (2016)	Congenital abnormalities, CHD, congenital cardiac defects, morphogenetic defect	Undefined
	Marbán-Castro et al (2021)	Congenital infection, congenital syndrome, congenital malformations, anomalies, abnormalities, foetal and child defects, birth anomaly Congenital Zika syndrome (CZS), (Zika-associated birth defect, congenital zika syndrome/infection	Undefined “A pattern of structural anomalies and functional disabilities secondary to central nervous system damage… whose most common clinical feature is microcephaly” “A spectrum of birth defects including foetal brain disruption sequence, brain anomalies, ocular anomalies, congenital contractures, intrauterine growth restriction, seizures, pyramidal or extrapyramidal abnormalities and neurodevelopmental delay”
	Brennan and Rayburn (2012)	Birth defects, congenital anomalies, congenital malformations, CHD	Undefined
	Williams and Chakravarty (2014)	Congenital anomalies, birth defects, congenital malformations, teratogenic exposure	“Congenital malformations or birth defects are alterations of morphogenesis of organs or body districts identifiable at birth” “A teratogenic exposure is one that has the potential to interfere with the normal functional or structural development of an embryo or fetus”
	Neal et al. (2009)	Congenital abnormalities, congenital defects	Undefined
	Munoz-Jordan (2017)	Birth defects, congenital abnormalities, Zika virus infections	Undefined
Congenital disorder(s)	van der Bom et al. (2011)	Congenital disorder Congenital malformation Congenital heart disease/defect (CHD)	Undefined Undefined “A gross structural abnormality of the heart or intrathoracic great vessels that is actually or possibly of functional significance”
	Barone et al. (2014) Freeze and Aebi (2005)	Genetic diseases Congenital anomalies CDG	Undefined Undefined “genetic diseases due to defective glycosylation of proteins and lipids” (Barone et al. 2014) “A family of genetic diseases due to defects in the synthesis of the glycan moiety of glycoproteins and glycolipids and in their attachment to proteins and lipids” (Barone et al. 2014) “A group of diseases that affect glycoprotein biogenesis” (Freeze and Aebi 2005)
	Willems et al. (2016)	CDG Genetic defects	“Caused by defects in the protein glycosylation pathway” Undefined
	Francisco et al. (2019b)	Inherited (human) disorders Genetic diseases, CDG	Undefined “CDG is a rapidly expanding genetic disease family with 100 types reported”
	Brasil et al. (2022)	CDG	“A group of rare genetic disorders affecting protein and lipid glycosylation and glycosylphosphatidylinositol anchor biosynthesis”
	Dennis et al. (2009)	CDG Polygenic diseases, complex traits, complex trait diseases	“Hypomorphic defects in N-glycan biosynthesis” Undefined
	Francisco et al. (2019a)	CDG	“A rapidly growing family of genetic diseases that currently include some 130 different types” “A large and very heterogeneous group of genetic disorders. They comprise defects in the synthesis of glycoprotein and glycolipid glycans, and their attachment to proteins and lipids”
	Kitazawa and Shima (2020)	Congenital disorder	“Hemophilia A is a congenital disorder caused by deficiency or malfunction of coagulation factor (F) VIII”
	Benigni et al. (2004)	Congenital disorders	Undefined
Congenital anomaly/anomalies	Parnell et al. (2017)	Congenital anomalies, congenital malformations, birth defects	Undefined
	Robinson et al. (2021)	Congenital toxoplasmosis Congenital anomalies, congenital infection, congenital disease	“An infection caused by the protozoan parasite Toxoplasma gondi”
	Malinowski et al. (2020)	Congenital anomalies Monogenic genetic disorder, congenital abnormalities, congenital disorder of glycosylation	“Structural or functional abnormalities usually evident at birth, or shortly thereafter, and can be consequential to an individual’s life expectancy, health status, physical or social functioning, and typically require medical intervention” Undefined
	Breech and Laufer (2009)	Congenital anomalies, congenital abnormalities	Undefined
	Kunisaki (2012)	Congenital anomalies, birth defects	“Congenital anomalies are the end products of aberrant organogenesis in utero. Some of the more common congenital anomalies encountered in neonatal intensive care units include diaphragmatic hernia, gastroschisis, oesophageal atresia, spinal bifida and heart defects”
	Bowman and McLone (2010)	Complex congenital anomaly/abnormality, birth defect, congenital defects	Undefined
	Bell et al. (2021)	Congenital anomalies Congenital malformations	“Any abnormality reported after birth” “Any “birth-defect” in pregnancies with and without live births whereas the remainder of the studies reported congenital anomalies only from live birth pregnancies”
	Abel et al. (2004)	Congenital anomaly	“Isolated sternal cleft is a rare congenital anomaly presents from birth to adulthood”
	Barqawi (2005)	Congenital anomalies, congenital abnormalities, foetal malformations, birth defects, congenital malformations	Undefined
	Weichert et al. (2011)	Congenital anomalies, congenital abnormalities	Undefined

^The 10 articles included for each key search term above are those with the highest number of different terms coded during analysis of each sub-set of 20 that were randomly selected for full-text evaluation

### Website review

The terminology used on the websites reviewed was notably less technical with less terms used than that in the peer-reviewed literature. The terms birth defect and congenital anomaly are most commonly used and often used interchangeably. Many websites did not define the term(s) used, and others offered different, conflicting definitions for the same term on different pages of the same website. Definitions of terms also varied between websites. See Table 3 and Table 4 for details.

**Table 3:** Summary of terms and definitions used by key international and national organisations (English-speaking countries) and initiatives involved in the birth defects field

Organisation/country	Classification	Aim/focus	Term and definition(s)	URL source
International organisations and initiatives				
World Health Organization (WHO)	WHO: United Nations Agency (Member States)	WHO works worldwide to promote health, keep the world safe, and serve the vulnerable	*Birth defects* are also known as *congenital abnormalities*, *congenital disorders or congenital malformations* Defined as *structural or functional anomalies* (for example, metabolic disorders) that occur during intrauterine life and can be identified prenatally, at birth, or sometimes may only be detected later in infancy, such as hearing defects. Broadly, congenital refers to the existence at or before birth *Congenital anomalies*: defined as *structural or functional anomalies* that occur during intrauterine life. Also called *birth defects, congenital disorders or congenital malformations*, these conditions develop prenatally and may be identified before or at birth, or later in life	https://www.who.int/news-room/fact-sheets/detail/birth-defects https://www.who.int/health-topics/congenital-anomalies#tab=tab_1
	WHO: International Statistical Classification of Diseases and Related Health Problems (ICD)	Provides critical knowledge on the extent, causes and consequences of human disease and death worldwide via data that is ICD reported and coded. Clinical terms ICD-coded are the basis for health recording and statistics on disease in primary, secondary, tertiary care, and on cause of death certificates	ICD-10: Chapter XVII: *Congenital malformations, deformations and chromosomal abnormalities*. Birth defects including inborn errors of metabolism, blood disorders of prenatal origin and single gene disorders also appear in other chapters ICD-11: *Developmental anomalies*: (1) primarily affecting one body system, 2) multiple development anomalies or syndromes, (3) chromosomal anomalies, excluding gene mutations, (4) conditions with disorders of intellectual development as a regular clinical feature, (5) other specified developmental anomalies, and (6) developmental anomalies, unspecified	https://icd.who.int/browse10/2016/en#/XVII https://icd.who.int/browse11/l-m/en#/http%3a%2f%2fid.who.int%2ficd%2fentity%2f223744320
	WHO: World Health Assembly (WHA) of member states	Decision-making body of WHO to determine WHO policies, appoint DG, supervise financial policies and review/approve the proposed programme budget	Resolution 63.17 (2010) *Birth defects: structural or functional abnormalities*, including metabolic disorders, which are present from birth. The terms birth defect and congenital disorders are used interchangeably	https://apps.who.int/iris/handle/10665/2378
International Clearinghouse for Birth Defects Surveillance and Research (ICBDSR)	NPO affiliated to WHO	Brings together birth defect programmes from around the world with the aim of conducting worldwide surveillance and research to prevent birth defects and to ameliorate their consequences	*Birth defects*: foetal and childhood conditions of prenatal cause	http://www.icbdsr.org/aboutus/
Global Birth Defects Website	Website established by International Committee for Congenital Anomaly Surveillance Tools as part of ZIKA Plan Project (EU funded)	Aims to strengthen birth defect surveillance and research, designed to bring birth defect (congenital anomaly) surveillance into mainstream public health activity	*Birth defects, congenital anomalies:* birth defects affect at least 3% of babies in most populations. A wide range of conditions are included: spina bifida, limb defects, cleft palate or lip, heart defects and Down syndrome being among the more common	https://globalbirthdefects.tghn.org/
Institute for Health Metrics and Evaluation (IHME) and Global Burden of Disease (GBD)	Research Institute (at University of Washington, USA)	*IHME*: research institute working in global health statistics and impact evaluation, in Seattle *GBD*: a tool to quantify BOD and health loss from hundreds of diseases, injuries and risk factors**,** to improve health systems and eliminate disparities	*Congenital birth defects* include livebirths (i.e. excludes stillbirths and pregnancy terminations) with any condition resulting from *abnormalities of embryonic development*, excluding minor anomalies as defined by EUROCAT and defects arising from infections or substance abuse	https://www.healthdata.org/results/gbd_summaries/2019/congenital-birth-defects-level-3-cause
Modell Global Database (MGDb)	Research collaboration	Estimates of the epidemiology and associated health burden of congenital disorders	*Birth defects, congenital disorders*: any potential pathological conditions arising before birth, whether evident at birth or manifesting later in life, including environmental congenital disorders, congenital malformations, single gene disorders, chromosomal disorders and disorders due to genetic risk factors. Using this definition, congenital disorders fall into two groups: (1) environmental congenital disorders (maternal exposure to infection, malnutrition or harmful environmental agents and disorders with principally endogenous causes) and (2) endogenous congenital disorders (chromosomal disorders, most congenital malformations, single gene disorders and disorders due to genetic risk factors)	https://datacompass.lshtm.ac.uk/id/eprint/2960/
Regional initiatives				
European network of population-based registries for the epidemiological surveillance of congenital anomalies (EUROCAT)	Regional (international) registry	Created to support data management for newborn health, birth defects and stillbirths	*Congenital anomalies*: (“All anomalies”)…*structural* malformations and chromosomal anomalies diagnosed in the foetus, baby or child *Other anomalies*: other congenital conditions, including congenital neoplasms (ICD10 C and D codes) and congenital endocrine (E), metabolic (E), immunologic (D) and haematologic (D) conditions	https://eu-rd-platform.jrc.ec.europa.eu/eurocat_en
WHO Regional Office for South-East Asia Newborn and Birth Defects Database (SEAR-NBBD)	Regional collaboration (SEAR)	An online integrated newborn-birth defect database to support data management for birth defects detected at birth, stillbirths and newborns in hospital settings	Birth defects: as per WHO definitions	https://www.who.int/southeastasia/sear-nbbd
European Network of Teratology Information Services (ENTIS)	Regional/global collaborative network of Teratology Information Services	To coordinate and collaborate the activities of the different Teratology Information Services (TIS) and collect and evaluate data in order to contribute to the primary prevention of birth defects and developmental disorders	*Teratogen*: an environmental agent such as a drug, chemical, virus or pollutant which interferes with the normal development of the embryo or foetus *Congenital anomalies, birth defects, developmental disorders*: not defined per se but inferred to result from teratogenic exposure defined above	https://www.entis-org.eu/
Organization of Teratology Information Specialists (OTIS)	NPO/professional scientific organization	Assesses and evaluates risks to pregnancy and breastfeeding outcomes from medications and other exposures	*Birth defects*: definition not specified	https://mothertobaby.org/about-otis/
Key fora and publications				
Birth defects surveillance: a manual for programme managers, 2nd Edition (2020)	Collaborative publication (WHO, CDC/NCBDDD, ICBDSR)	A tool for development, implementation and improvement of a congenital anomaly surveillance programme, particularly for countries with limited resources	*Congenital anomalies*, also known as *birth defects*, are *structural or functional abnormalities*, including metabolic disorders, which are present from birth. Congenital anomalies are a diverse group of disorders of prenatal origin, which can be caused by single gene defects, chromosomal disorders, multifactorial inheritance, environmental teratogens and micronutrient malnutrition	https://www.cdc.gov/ncbddd/birthdefects/surveillancemanual/chapters/objectives.html
International Conference on Birth Defects and Disabilities in the Developing World (ICBD)	Biennial conference	Exchange of knowledge, experience, best practices globally to promote coordinated, effective, transparent global and national action on birth defects	*Birth defects, congenital disorders*: abnormalities in *structure or function* present from birth	https://www.marchofdimes.org/our-work/icbd-conference Darmstadt et al. (2016)

**Table 4:** Summary of terms and definitions used by key national organisations and initiatives in English-speaking countries involved in the birth defect field (as of September 2022)

Australia				
Healthdirect Australia	*NPO (government-owned)*	National virtual public health information service working towards the health priorities of Commonwealth, state and territory funders to improve the health of Australians	*Birth defects*: affect how the body looks and functions, such as structural problems with bones, skin, nerves or tissues. Examples include *cleft lip*, *spina bifida* and *neural tube defects*. Birth defects can also cause mental disabilities	https://www.healthdirect.gov.au/birth-defects https://about.healthdirect.gov.au/
Western Australian Register of Developmental Anomalies (WARDA)	Registry (sub-national)	A record of babies and children diagnosed with developmental anomalies > 6 years	*Developmental anomalies*: a broad term for conditions present at conception or occurring before the end of pregnancy. Developmental anomalies are also sometimes called *birth defects*, *congenital malformations* or *congenital anomalies*	https://www.healthywa.wa.gov.au/Articles/U_Z/Western-Australian-Register-of-Developmental-Anomalies
South Australian Birth Defects Register (SABDR)	Registry (sub-national)	Collects information on all children born in South Australia with a significant birth defect detected in first 5 years of life	*Birth defect*: any abnormality, *structural* or f*unctional*, identified up to the child’s fifth birthday, provided that the condition had its origin before birth	https://www.wch.sa.gov.au/professionals/registers/birth-defects-register
Australian Birth Defects Society	NPO	Aims to inform both the public and medical professionals about the latest news, academic findings, facts and advice for pregnant women and families	*Birth defect*: a problem that develops in utero (the womb) during pregnancy. Also referred to as *congenital anomalies, congenital malformations, congenital abnormalities or congenital disorders*. May be minor or significant (some can lead to death or major health complications)	https://www.birthdefectssociety.org.au/what-are-birth-defects
Genetic Alliance Australia	NPO	Facilitates support for those affected directly/indirectly by genetic conditions throughout Australia (improve quality of life of patients and families affected by genetic conditions/rare disease)	*Genetic condition*: a gene or chromosome fault can result in a genetic condition. Genes are the instructions that determine physical characteristics such as height, hair and eye colour, strength of our bones and the correct functioning of our bodies. Genes are located on chromosomes inside the cells of our bodies. A genetic condition can run in families but may occur without a previous family history	https://www.google.com/search?client=firefox-b-d&q=Genetic+alliance+australia
Canada				
Health Infobase	Government	Birth defects and congenital anomalies	*Congenital anomalies also known as birth defects, congenital disorders or congenital malformations* are *usually structural or functional in nature*. They are identified prenatally, at birth, or sometimes later in infancy. Approximately 50% of all congenital anomalies cannot be linked to a specific cause; however, there are some known risk factors which include genetics and environmental contaminants	https://health-infobase.canada.ca/congenital-anomalies/
Canadian Congenital Anomalies Surveillance System (CASS)	Registry (government)	Population-based surveillance of congenital anomalies which captures secondary health surveillance information on all infants born in Canada and diagnosed as having a congenital anomaly within the first year of life	*Congenital anomalies*: definitions as above	https://www.canada.ca/en/public-health/corporate/mandate/about-agency/access-information-privacy/canadian-congenital-anomalies-surveillance-system-ccass-march-2016.html
New Zealand				
New Zealand Congenital Anomalies Registry (NZCAR) formerly NZ Birth Defects Registry	Registry	Provides reliable, valid data on prevalence of birth defects in New Zealand; monitors prevalence (e.g. over time, across areas, and between demographic groups); investigates clusters; uses data to assess effectiveness of prevention programmes (e.g. screening); provides advice to HCP and provides data and analysis for planning of health services and development of related health policy.	*Congenital anomalies*: no definition provided	https://www.ehinz.ac.nz/projects/new-zealand-congenital-anomalies-registry/
Health Navigator	NPO	Provide reliable and trustworthy health information and self-care resources	*Birth defect*: a problem present at birth that happens when a baby is developing in their mother’s body. Most happen in first 3 months of pregnancy when organs are developing. A birth defect may affect the way the body looks, works or both. Some birth defects (such as cleft lip) are easy to see, but others (such as heart defects or hearing loss) are found using special tests. Birth defects can vary from mild to severe	https://www.healthnavigator.org.nz/health-a-z/b/birth-defects/
UK				
UK Health Security Agency (UKHAS)	Executive agency sponsored by Department of Health and Social Care	Responsible for protecting every member of every community from the impact of infectious diseases, chemical, biological, radiological and nuclear incidents and other health threats. Provides intellectual, scientific and operational leadership at national and local level, and globally to make the nation’s health secure.	Congenital anomalies, birth defects: no definition provided but refers to NCARDRS	https://ukhsa.blog.gov.uk/2018/03/03/the-work-we-are-doing-on-birth-defects/
Newlife Birth Defects Research Centre (BDRC)	Research centre (Institute of Child Health, University College London)	Centre of excellence for genetics and developmental biology research into clinically important birth defects. Enhances translation of research findings into new diagnostics and therapies and assesses outcomes on a population level. Trains next generation of birth defect research investigators	*Birth defects* are a leading cause of infant mortality in the Western world. One in every 40 pregnancies in Europe is affected by a birth defect, of which there are more than 4,000 types. Some of the commoner birth defects include spina bifida, congenital heart defects, congenital deaf-blindness, cleft lip and palate and Down’s syndrome	https://www.ucl.ac.uk/child-health/research/developmental-biology-and-cancer/newlife-birth-defects-research-centre
National Congenital Anomaly and Rare Disease Registration Service (NCARDRS)	Surveillance registry (government)	NCARDRS records those people with congenital abnormalities and rare diseases across the whole of England	*Congenital anomalies*: present at delivery, originating before birth, and include *structural, chromosomal and genetic conditions*. Some congenital anomalies are detectable during pregnancy, and others are not	https://www.gov.uk/guidance/the-national-congenital-anomaly-and-rare-disease-registration-service-ncardrs#congenital-anomalies https://www.gov.uk/government/publications/ncardrs-congenital-anomaly-annual-data/ncardrs-statistics-2019-summary-report
Genetic Alliance UK	Member alliance	An alliance of over 200 patient organisations with a mission to ensure that high quality services, information and support are provided to all	Genetic, rare and undiagnosed conditions	https://geneticalliance.org.uk/
USA				
Centers for Disease Control and Prevention (CDC), USA	National public health federal agency (government)	Resources on birth defects including key information on US birth defect prevalence statistics, prevention of birth defects and multi-media resources, tracking logic models, surveillance guidelines and specific clinical information on a range of birth defects	*Birth defects: structural* changes present at birth that can affect almost any part or parts of the body (e.g. heart, brain and foot). They may affect how the body looks, works, or both. Birth defects can vary from mild to severe	https://www.cdc.gov/ncbddd/birthdefects/index.html
National Birth Defects Prevention Network (NBDPN)	Voluntary organisation (member-based umbrella registry)	To establish/maintain national network of state/population-based programmes for birth defects surveillance and research	*Birth defects*: abnormal conditions that happen before or at the time of birth. Some are mild–like an extra finger or toe. Some are very serious—like a heart defect. They can cause physical, mental or medical problems. Some (Down syndrome or sickle cell anaemia) are caused by genetic factors. Others are caused by certain drugs, medicines or chemicals, and causes of most birth defects are still a mystery	https://www.nbdpn.org/ https://www.nbdpn.org/docs/Pamplet_BDprevention.pdf
March of Dimes (MOD)	NPO	To end preventable maternal health risks and death, preventable preterm birth and infant death and closing the health equity gap	*Birth defects*: *structural changes* that are present at birth. They may affect how the body looks works or both Health conditions that are present at birth. They change the *shape or function* of one or more parts of the body Birth defects are *physical or chemical abnormalities* that are present at birth	https://www.marchofdimes.org/complications/birth-defects-and-your-baby.aspx

## Discussion

The aim of this study was to demonstrate the variety of collective terms and definitions related to congenital disorders used in both the peer-reviewed literature and on websites of key international and national initiatives. The review results show a wide variety of different terms in use, often interchangeably, in both settings. Definitions are rarely provided, and in some cases, particularly in the website review, different definitions are used interchangeably, for example, on the WHO website. Three specific terms, congenital anomal/anomalies, birth defects and congenital disorders, were also further evaluated following recommendations related to these terms in 2006 (World Health Organization 2006), and results indicated poor uptake of terms recommended for use. The inconsistent use of terms and definitions highlights the need for development of defined, uniform terms and consistent implementation of both terminology and definitions.

### Previous consensus efforts

The MOD and WHO made a concerted effort to achieve global consensus on birth defect terminology in 2006, when a meeting involving 18 global experts from both LMIC and HIC was convened following the publication of the March of Dimes global report on birth defects (Christianson et al. 2006). One aim of this WHO/MOD meeting was to reach agreement on the definition of terms related to congenital disorders (Christianson et al. 2006, World Health Organization 2006).

Participants at the meeting achieved consensus, proposing synonymous use of the terms “birth defects” and “congenital disorders” (World Health Organization 2006). Estimates of congenital disorders for 190 countries published in the MOD global report (Christianson et al. 2006) were also endorsed at the meeting, with data differences between these and WHO childhood death estimates for congenital anomalies thought to be, in part, due to the difference in terminologies used (World Health Organization 2006). MOD representatives preferred the broader and more accepted term birth defects, defined as “abnormalities in structure or function, including metabolism, which are present from birth” (World Health Organization 2006)—recognising that some conditions are obvious at birth while others manifest later in life. While this term was preferred due to its broad use and acceptance, MOD acknowledged that it is not universally accepted (World Health Organization 2006). They also suggested grouping according to aetiology including as follows:

- Genetic disorders, including single gene defects and chromosomal abnormalities
- Partially genetic disorders, including multifactorial congenital malformations
- Non-genetic disorders, due to abnormalities of the foetal environment, including teratogens, mechanical forces (e.g. constraint), vascular accidents and unknown causes (Christianson et al. 2006)

In contrast, WHO representatives preferred the term “congenital disorders” (World Health Organization 2006). MOD/WHO representatives advised against the use of the term congenital *anomalies*, as described in the 10th revision of the International Classification of Diseases (ICD-10) (Shibuya and Murray 1998, World Health Organization 1992, 2006). The ICD codes are used extensively to gather and exchange data on birth defects and for assigning cause of death. However, this sub-set of disorders excludes “functional birth defects including non-syndromic, congenital disability…common single gene disorders… inborn errors of metabolism” and “many teratogen-induced birth defects, including congenital syphilis, congenital rubella syndrome and iodine deficiencies (World Health Organization 2006). Today this would include TORCH congenital infections, including *Toxoplasma gondii*, other agents, rubella, cytomegalovirus (CMV) and herpes simplex virus (HSV). Collectively, these excluded conditions account for more than a third of congenital disorders in ICD-10 (Christianson and Modell 2004; Malherbe et al. 2016; Modell et al. 2016).

A further recommendation from the 2006 MOD meeting was for the WHO ICD team to engage relevant organisations to develop a “universally acceptable classification of birth defects”. The most recent revision, ICD-11, has implemented an updated, more sophisticated structure and invited evidence-based proposals to promote openness and transparency—which has included the incorporation of inputs relevant to congenital disorders and the use of the collective term “developmental anomalies” (World Health Organization 2019, 2022, Drösler et al. 2021, Harrison et al. 2021).

The consensus achieved at the 2006 WHO/MOD meeting was thought to have ended “over two decades of uncertainty” (World Health Organization 2006). However, the recommendations were not widely publicised in other formats or to other audiences, and the evidence indicates little uptake of uniform, consistent terminology globally (Modell et al. 2016), both in the literature and websites reviewed. This may also account for the continued preferred use of the term congenital anomaly/anomalies observed in both reviews.

### General observations

This review shows that despite the recommendations of the 2006 MOD/WHO meeting (World Health Organization 2006), there is still no global consensus on birth defect terminology. This may be partly because sustained advocacy and awareness around congenital disorders have waned in recent years due to resource restrictions and changes in focus (e.g. shift in focus by MOD to preterm births), despite the increasing proportion of child deaths related to these disorders. This has resulted in congenital disorders being under-prioritised within the broad mandate of many health organisations.

#### Community genetic services

The historic challenge of finding an appropriate “home” for congenital disorders has also contributed to loss of momentum in the field of community genetics. The unique characteristics of congenital disorders, including diverse natural histories, varying impact across the lifespan and diverse aetiologies, make them difficult to place. Little traction has been gained by placing these conditions under the umbrella of non-communicable diseases (NCDs)—where almost exclusive focus remains upon adult-onset disorders. A more suitable placement for congenital disorders, particularly early onset conditions, may be within maternal, child, adolescent and reproductive health programmes, rather than NCDs—but this requires further discussion and debate within the global public health community.

Historically, governments began to recognise the need for community genetic services once the infant mortality rate (IMR) dropped below 40–50 per 1 000 live births—and significant further reductions could only be achieved by comprehensively addressing congenital disorders (Modell and Kuliev 1998; Christianson and Modell 2004, World Health Organization 1999). In many LMIC, the need for community genetic services is being recognised later due to the continued burden of infectious diseases of childhood and as NCDs gain increasing recognition (Kerber et al. 2013). In the interim, many of those affected by congenital disorders do not receive a diagnosis or the care required after diagnosis. This is contrary to the underlying philosophy of the Sustainable Development Goals (SDG) “to leave no one behind” and the 2030 SDG3 target to “end preventable deaths of newborns and children under five years of age” (United Nations 2015). It also echoes the consensus of the 2015 International Conference on Birth Defects and Disabilities in the Developing World (ICBD) that “children with congenital disorders remain left behind in policies, programmes, research and funding” (Darmstadt et al. 2016; Modell et al. 2018). For true inclusivity, global efforts must incorporate integrated programmes combining care and prevention, on the principle that “care is an absolute and prevention is the ideal” (Christianson et al. 2000).

#### Endogenous versus environmental congenital disorders

The need to distinguish between environmental congenital disorders caused by maternal exposure to teratogens (alcohol, recreational and prescription drugs, tobacco, occupational exposure, infection malnutrition), and congenital disorders arising from endogenous causes, was recognised both by MOD at the 2006 joint meeting with WHO (World Health Organization 2006) as outlined above and during the subsequent development of the MGDb. This clear distinction has not been made by other key initiatives or programmes but has important implications for policy development and implementation. The majority of environmental congenital disorders are preventable and may even be eliminated through existing, traditional public health measures such as immunisation, food fortification, sanitation and community information. However, this does not apply to disorders with internal (endogenous) causes such as most congenital malformations, chromosomal disorders, single gene disorders and disorders due to common genetic risk factors (Modell et al. 2016). The endogenous disorders^4^ included in the MGDb may be treatable or correctable, or life-limiting and incurable, and present health services providers, especially those in LMIC, with a difficult challenge. It is proposed that the terms endogenous and environmental congenital disorders are more widely used to differentiate between these aetiologies.

### Implications for data sharing and policy development

The inconsistent use of undefined terms makes it difficult to compare data sets both within and between populations, inviting the reader to make assumptions based on previous knowledge and experience which may differ from that intended by the authors. Some established registries, such as the umbrella registry EUROCAT, enable valid comparisons by clearly defining a core dataset to be collected by all participating registries, supported by detailed guidelines for case reporting and ascertainment (EUROCAT 2018). In other regions and countries, especially LMIC where registries are under-developed or non-existent and there is a lack of detail on congenital disorders, terms and definitions are often adopted *ad hoc* with little or no evidence-base and bundling and grouping for presentation to policymakers is often unplanned and unstandardised.

The provision of standardised, accessible and reliable output data for congenital disorders associated with structural abnormality (ICD-10 Chapter XVII) by congenital anomaly registries may account for the extensive use of, and preference for, the term congenital anomalies across both the scientific literature and public websites. Unfortunately, as programmes expand to include additional, less obvious, functional congenital disorders, the terminology often remains unchanged, leading to difficulties in comparing data from different sources. Without standardisation to ensure accuracy, comparisons across and between countries and regions will not be possible and some important conditions will remain unrecognised and underserviced by health services.

## Limitations of the study

A robust scoping review methodology was employed in this study, and 20 articles were evaluated per specific search term due to resource constraints and the high number of articles identified. Similarly, the website review was preliminary, with the inclusion of a single source for a term and definition used by an individual entity. Both reviews in this study were limited in scope to the English language and in the number of studies that could be evaluated due to resource constraints. Nevertheless, the results revealed the range of terms and definitions used globally. Support for the call for standard terminology and definitions will be enhanced by a broader search which also includes additional languages.

## Recommendations for future standardisation efforts

### Tailoring of terms for specific audiences

Terminology development and usage should consider the audience being addressed. As demonstrated by the use of different terms in the literature as compared to key websites in this review, different audiences require and prefer the use of specific terms, such as “congenital disorders of glycosylation” identified for papers related to this specific field (Freeze and Aebi 2005). The scientific literature is mainly aimed at specialists, but many other target audiences are multidisciplinary, including a combination of public health directors, primary health care practitioners, politicians and policymakers, the media and patient support associations with varying levels of genetic expertise. If genetic information and services are to be made available to all who may benefit, then agreed, clear and simple terms must be used and pitched appropriately for a general audience. This would both facilitate communication among health professionals working in different disciplines and support them in providing correct and consistent information to others.

The understanding of the lay population, including patients, patient organisations and the general public, is equally important. Some terms may have negative connotations or be considered derogatory by those living with congenital disorders, as highlighted by Mai et al. (2012) in relation to the use of birth “defects”. While the present review focused solely on English terminology, translation into vernacular language requires careful consideration since a suitable term in one country may not be deemed appropriate in another.

### Role of international organisations

Key international and regional initiatives working in the fields of congenital disorders and community genetics should address the issue of terminology and lead by example through collaborating on the global harmonisation of uniform terms and their implementation. Such organisations have an opportunity to set the tone through the development and consistent implementation of uniform terms for congenital disorders, underpinned by a companion glossary.

### Need for wider consultation

Recent work undertaken by the global rare disease community and sponsored by Rare Diseases International (RDI) to develop operational definitions for these conditions offers insight into the development of uniform terms and definitions for congenital disorders. A group of 18 experts from six continents participated in a series of three online workshops and two surveys to review and reach agreement on key statements related to rare diseases. The group agreed that the operational definition of rare diseases “must be clear and practical to implement, with defined parameters that permit a uniform, unambiguous, objective interpretation of the definition by a wide range of stakeholders across the world” (Rare Diseases International 2022). The results of this work have been actively and widely shared across a range of stakeholders (Rare Diseases International 2022) and in a forthcoming scientific publication (Wang et al., unpublished).^5^

Recommendations for future work to develop and advocate for standard definitions include the following:

- Agreement on terms and definitions that are precise, comprehensible and uniform across the full spectrum of congenital disorders. Such terms should be transparent and accurate in their clinical implications and also reflect the changes made in ICD-11.
- Agreed terms should be sensitive to the preferences and tailored to the pitch, of the users, especially patients and lay-audiences. To ensure effective communication to both specialist and lay audiences, two-tiers of terms could be considered to meet the needs of both the specialist and non-specialist, e.g. haemoglobinopathies/haemoglobin disorders.
- Use of developed terms should be accompanied by the definition to optimise their understanding by the reader, minimise ambiguity and promote consistent implementation.
- Terms and definitions could be condensed into an online, WHO-endorsed glossary for global dissemination.
- Implementation of a robust, transparent consultative process followed by wide dissemination of the results in a variety of formats to promote optimal uptake.

## Conclusion

The quantity and diversity of terms used to describe congenital disorders and the lack of standardised definitions highlight the need for concerted, international efforts to develop uniform terms for consistent use. This is a critical issue, both in LMIC where empirical data on congenital disorders is lacking and also in the HIC that host key funding sources and major research centres. To ensure that those affected by congenital disorders are not left behind, they must be diagnosed and counted. For this to be undertaken comprehensively and accurately, the components of this burden of disease must be clearly defined in a language understood by all.

## Supplementary Information

Below is the link to the electronic supplementary material.

Supplementary file1 (XLSX 22 KB)

## Footnotes

1 Malaria infection strongly influences the birth prevalence of haemoglobin disorders and G6PD.

2 Community genetics is defined as “the art and science of the responsible and realistic application of health and disease-related genetics and genomics knowledge and technologies in human populations (communities) to the benefit of individual persons.” (Ten Kate et al. 2010).

3 Wild card (*) used in search phrase with root words in PubMed search to identify all suffix variations of terms.

4 The term “constitutional” has been replaced by “endogenous” in a recent application of the MGDb in South Africa (Malherbe et al. 2021) with regard to conditions with a genetic/partially genetic aetiology and may be more preferable for future use.

5 Wang C, Whiting A, Rath A, et al. A reference to improve the recognition and visibility of rare diseases (Submitted: Orphanet Journal of Rare Diseases, March 2023).
